# The incidence rate and gene mutation characteristics of hyperphenylalaninemia in Yunnan Province, Southwest China

**DOI:** 10.1186/s13023-025-04114-3

**Published:** 2025-11-25

**Authors:** Qiong Wang, Jiang Duan, Xiaolong Zhao, Zhiye Qi

**Affiliations:** 1 Newborn Screening Center, First Affiliated Hospital of Kunming Medical University, Kunming, Yunnan Province China; 2 Department of Pediatrics, First Affiliated Hospital of Kunming Medical University, Kunming, Yunnan Province China; 3 Children’s Medical Center, First Affiliated Hospital of Kunming Medical University, Kunming, Yunnan Province China

**Keywords:** Hyperphenylalaninemia, Neonatal screening, Genotype, Phenotype, Incidence rate

## Abstract

**Background:**

The global incidence of Hyperphenylalaninemia (HPA) demonstrates significant geographical variations, exhibiting distinct regional and ethnic characteristics in both phenotypic manifestations and genotypic profiles. To date, there remains a paucity of data regarding the genotype-phenotype correlation in pediatric patients with phenylalanine hydroxylase deficiency (PAHD) from Southwest China. This study aims to conduct a retrospective analysis of neonatal HPA prevalence and characterize *PAH* gene mutations in Yunnan Province in Southwest China. These findings are expected to establish an evidence base for optimizing clinical follow-up protocols, facilitating genetic counseling, and enabling prenatal molecular diagnosis for affected children.

**Methods:**

From January 2013 to December 2023, neonatal screening data for HPA were retrospectively collected from the Yunnan Neonatal Screening Center. Neonates with presumptive positive screening results underwent confirmatory diagnosis through quantitative analysis of phenylalanine levels using tandem mass spectrometry. Subsequently, HPA-related genetic variants were identified by next-generation sequencing technology. Putative pathogenic mutations detected in probands were validated through Sanger sequencing of trios.

**Results:**

A total of 1,261,043 newborn screening samples for phenylalanine were analyzed, with 125 cases confirmed as HPA. The overall incidence rates were 0.99 per 10,000 for HPA, 0.98 per 10,000 for PAHD, and 0.16 per 100,000 for tetrahydrobiopterin deficiency. Genetic analysis of hyperphenylalaninemia-related genes was performed in 84 children, revealing 49 *PAH* variants, 2 *PTS* variants, and 1 *QDPR* variant, with a total of 164 mutation sites identified. Missense mutations constituted the predominant variant type. The most frequent *PAH* mutations were c.728G > A/p.R243Q (26.88%), c.331C > T/p.R111* (9.38%), c.320A > G/p.H107R (8.13%), c.158G > A/p.A53H (7.50%), and c.441 + 2T > A/splicing (5.00%), with clustering observed in exons 7, 11, 6, and 3. A novel *PAH* mutation (c.60 + 4A > G/ p.?.) was identified.

**Conclusions:**

The incidence and genetic mutation spectrum of HPA in Yunnan Province, Southwest China, exhibit distinctive characteristics when compared with other regions in China and international reports. These differences may be attributed to the unique geographical distribution of HPA patients and ethnic-specific genetic characteristics in this region. Furthermore, specific genetic mutations demonstrate potential associations with clinical phenotypes in PAHD. The novel *PAH* mutations identified in this study have expanded the current *PAH* gene database.

## Background

Hyperphenylalaninemia (HPA) is a genetic metabolic disorder affecting phenylalanine metabolism, inherited in an autosomal recessive manner, involving phenylalanine hydroxylase (PAH) deficiency and its coenzyme tetrahydrobiopterin deficiency (BH4D) [1]. Phenylalanine hydroxylase deficiency (PAHD) is caused by mutations in the *PAH* gene resulting in reduction or loss of PAH enzyme activity, and lead to high concentrations of phenylalanine in blood and cerebrospinal fluid [2]. The clinical manifestations of PAHD range from mild hyperphenylalaninemia (mHPA) that requires no treatment to classical phenylketonuria (cPKU) that necessitates strict control of phenylalanine intake. This phenotypic variation may be associated with the extent of PAH activity reduction caused by different genotypes [3]. BH4D accounts for approximately 1 ~ 2% of cases of HPA [4], but BH4D not only results in an elevated blood phenylalanine concentration, but also in a reduction of neurotransmitters like dopamine and serotonin, ultimately causing severe neurological symptoms and signs, and even fatal outcomes [5].

Currently, many countries worldwide have implemented neonatal screening for HPA [6]. Results of epidemiological studies suggest that the global incidence of PAHD is approximately 0.64/10,000 individuals, with significant variations across different regions [7]. The incidence of PAHD in Southeast Asia is 0.03/10,000, while that in the Middle East/North Africa reaches 1.18/10,000 [7]. In China, the incidence of PAHD is 0.68/10,000 [8]. Furthermore, it exhibits distinct regional and ethnic characteristics in both phenotype and genotype [9–11]. The most common *PAH* gene mutation is p.R408W in the world [12]. In China, p.R243Q is the most prevalent mutation in the *PAH* gene [13], whereas p.A243G is the most common one in northern China [14]. *PAH* mutation spectrum analysis and phenotypic evaluation are crucial for the clinical diagnosis and treatment of children with PAHD. Since 2012, a government-supported free neonatal screening program has been launched in Yunnan Province, Southwest China. This program has increased the screening rate for neonatal inherited metabolic diseases, enabling many neonates with HAP to receive early diagnosis and treatment. But there are no relevant reports on the genotype, phenotype, and their relationship in children with PAHD in Southwest China. In this study, we gathered phenylalanine screening data from newborns in Yunnan Province spanning the past decade. Through analyzing the phenotypes of PAHD patients and the genotypes of *PAH* mutations, we summarized the mutation and phenotype spectrum of PAHD. For the first time, we reported the mutation spectrum of the *PAH* gene in children with PAHD in Southwest China and identified a novel *PAH* gene mutation site.

## Materials and methods

### Research subjects and phenotypic classification

Study subjects were newborns who underwent phenylalanine screening at the Neonatal Screening Center of the First Affiliated Hospital of Kunming Medical University between 2013 and 2023. Neonatal screening for HPA employs the time-resolved immunofluorescence assay with the Wallac 1420 analyzer (PerkinElmer Inc., Waltham, MA, USA) to determine the phenylalanine concentration in dried blood spot samples. For the definition of presumptive positive screening results, a strict threshold of phenylalanine concentration ≥ 2 mg/dL was established; this cutoff was determined based on clinical diagnostic needs and alignment with common neonatal screening standards to balance sensitivity and specificity. All samples with phenylalanine levels reaching or exceeding this threshold were categorized as presumptive positive and further subjected to confirmatory testing. Confirmatory diagnosis of HPA was performed via tandem mass spectrometry (MS/MS) utilizing the AB SCIEX system (AB SCIEX LLC, Framingham, MA, USA). To ensure consistency with screening results and diagnostic accuracy, the confirmatory positive threshold for phenylalanine was set at ≥ 120 µmol/L. For the verification of assay precision, analytical performance evaluation was conducted, and the intra-batch coefficient of variation (CV) and inter-batch CV for phenylalanine quantification were both maintained below 10%, thereby ensuring the reliability of HPA confirmatory results.

Children with PAHD are categorized into classic phenylketonuria(cPKU, serum phenylalanine concentration: ≥1200 µmol/L), mild phenylketonuria (mPKU, serum phenylalanine concentration: 360 ~ 1200 µmol/L), and mild hyperphenylalaninemia (mHPA, serum phenylalanine concentration: 120 ~ 360 µmol/L) based on their highest serum phenylalanine concentration prior to dietary treatment [15]. Informed consent was obtained from the guardians of the newborns, who signed the corresponding consent form. This study was approved by the Ethics Committee of the First Affiliated Hospital of Kunming Medical University.

### Gene mutation detection

Samples of 4 ml of peripheral venous blood were collected from children with HPA and their parents, and immediately transferred to ethylenediaminetetraacetic acid-anticoagulant tubes. Genomic DNA was extracted from the blood samples using a column chromatography method. Subsequently, a targeted gene panel was used to sequence the exon coding regions of HPA-related genes (including *PAH*,* PTS*,* QDPR*,* GCH1*,* PCBD1* and *DNAJC12)* on the Illumina HiSeq high-throughput sequencing platform. The average sequencing depth of the target regions in exome sequencing was ≥ 100×, with 96% of the target captured regions having a sequencing depth greater than 20×. To systematically analyze large deletions and duplications across all cases, the SALSA^®^ MLPA^®^ Probemix P055 *PAH* Reagent Kit (MRC-Holland, Amsterdam, the Netherlands) was employed to detect such structural variants in the *PAH* gene. Positive results from MLPA were further verified using quantitative Real-Time PCR. For children with no detected point mutations or large deletions in the *PAH* gene, whole-genome sequencing was performed to detect *PAH* gene mutations, so as to clarify whether there are regulatory variants in the non-coding regions and complex structural variants of the *PAH* gene. Compare these sequences with the reference sequence (UCSC hg19) to identify possible gene mutations. The pathogenicity rating and data interpretation rules for variant loci are based on the guidelines of the American College of Medical Genetics and Genomics (ACMG) and the application recommendations of the Sequence Variant Interpretation (SVI) expert panel to the guideline standards [16]. Specifically, variant-specific evidence was systematically collected and categorized into five classes (Pathogenic, Likely Pathogenic, Uncertain significance, Likely Benign, Benign) based on the guideline’s defined evidence criteria. The final pathogenicity classification was recorded with clear mapping to the ACMG evidence codes (e.g., “P: PVS1 + PS3 + PM2”) to ensure traceability. Mutant loci with a frequency greater than 1% in databases such as 1000 Genomes, ExAC, and gnomAD, as well as non-functional variant loci, are excluded. Candidate gene variant loci are identified through pathogenicity prediction (using software such as SIFT, Polyphen2, CADD, Mutation Assessor, NetGene2 Server), clinical symptom comparison, querying relevant disease databases (ClinVar, HGMD, PubMed, etc.), and literature reference. Sanger sequencing technology was utilized to further verify the mutation sites in the family samples.

According to the Phenylalanine Hydroxylase Gene Variant Database, genetic variants in the *PAH* gene are classified into synonymous mutations, missense mutations, nonsense mutations, frameshift mutations, and splice site mutations. To analyze the relationship between mutation types and phenotypes, the variants were further categorized into null mutations and missense mutations [17]. The genotypes of children with PAHD were divided into null/null, null/missense, and missense/missense.

### Statistical analysis

Statistical analyses were performed using IBM SPSS Statistics version 26.0. The comparison of rates among multiple groups was conducted using the chi-square test and Fisher’s exact probability method, while Kruskal-Wallis rank-sum test was utilized to analyze phenotypic differences among children with different genotypes. A *p*-value < 0.05 was considered as statistically significant.

## Results

### HPA incidence rate and clinical classification

A total of 1,261,043 neonatal samples underwent phenylalanine screening, achieving a screening rate of 98.21%. Among these samples, 125 cases of HPA were confirmed, comprising 60 males and 65 females. During the follow-up period, the phenylalanine concentration of one case of mHPA increased to 372.0 µmol/L at 6 months of age, prompting the administration of dietary therapy. The final diagnosis revealed 31 cases of cPKU (24.80%, 31/125), 45 cases of mPKU (36.00%, 45/125), 47 cases of mHPA (37.60%, 47/125), and 2 cases of BH4D (1.60%, 2/125). The average blood phenylalanine levels were 1660.29 µmol/L, 730.39 µmol/L, 200.81 µmol/L, and 1124.5 µmol/L, respectively (Table 1). There was no statistically significant difference in the incidence of various types of HPA between male and female patients (χ2 = 0.779, *P* = 0.399). Both of the 2 children with BH4D underwent BH4 loading test and urinary pterin profile analysis. At 24 h after BH4 administration, their blood phenylalanine levels decreased by 36.3% (from 18.2 to 11.6 mg/dl, with *QTPR* gene mutation) and 74.7% (from 8.23 to 2.08 mg/dl, with *PTS* gene mutation) respectively, compared with those before medication. No obvious abnormalities were observed in the urinary pterin profile analysis. The overall incidence rate of HPA is 0.99/10,000, with the incidence rate of PAH deficiency being 0.98/10,000 and that of BH4D being 0.16/100,000.

### Mutation spectrum and allelic frequency distribution of the HPA-related gene

HPA-related gene testing was completed in 84 patients. Among them, there were 59 cases of Han Chinese and 25 cases of ethnic minorities, 14 exhibited homozygous mutations, 66 had compound heterozygous mutations, and 4 were found to have one mutation site. Specifically, there were 2 mHPA cases, 1 mPKU case, and 1 cPKU case (Table 1).

**Table 1:** Clinical classification and genetic testing results of HPA patients [n (%)]

	cPKU	mPKU	mHPA	BH4D	Total
Case Number	31(24.80)	45(36.00)	47(37.60)	2(1.60)	125
Peak Phenylalanine value(µmol/L)	1660.29	730.39	200.81	1124.5	-
Number of genetic tests	24(28.57)	27(32.14)	31(36.90)	2(2.38)	84
Homozygote	8(57.14)	4(28.57)	1(7.14)	1(7.14)	14
Compound heterozygote	15(22.73)	22(33.33)	28(42.42)	1(1.52)	66

**Fig. 1:**
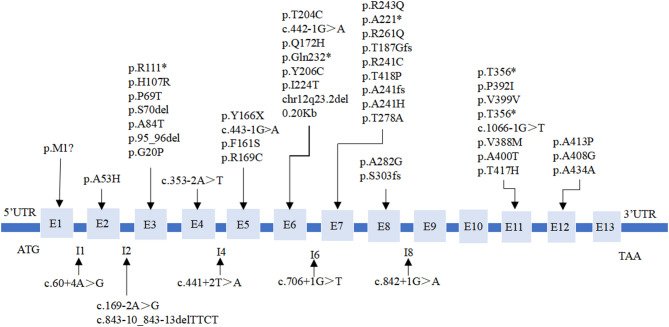
Mutation types of *PAH* gene in PAHD patients

A total of 164 gene mutation sites were detected, encompassing 49 types of *PAH* gene mutations (Fig. 1), 2 types of *PTS* gene mutations, and 1 type of *QDPR* gene mutation. Among these, there were 28 missense mutations (53.85%, 28/52), 10 splice mutations (19.23%, 10/52), 7 nonsense mutations (13.46%, 7/52), 5 frameshift mutations (9.62%, 5/52), and 1 synonymous mutation (1.92%, 1/52). In a case of cPKU patient, a 0.2 kb fragment heterozygous deletion involving the *PAH* gene was detected (1.92%, 1/52). The mutation sites of the *PAH* gene were primarily concentrated in exons 7 (18.37%, 9/49), 11 (16.33%, 8/49), 6 (14.29%, 7/49), and 3 (14.29%, 7/49) (Table 2). The *QDPR* gene mutation is located on chromosome 4p15.3, exon 7, and is a homozygous mutation c.661C > T/p.R221X, with an allele frequency of 1.22% (2/164). The *PTS* gene mutation is located on chromosome 11q22.3, exon 5, and consists of compound heterozygous mutations c.286G > A/p.D96N and c.259C > T/p.P87S, with an allele frequency of 0.61% (1/164). No other gene mutation sites related to BH4D such as *GCH1*,* PCBD1* and *DNAJC12* were detected.

**Fig. 2:**
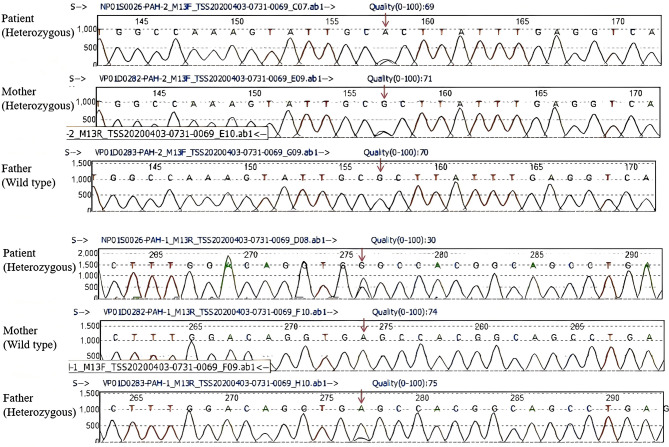
Sanger sequencing of the child with novel *PAH* gene mutation site. The red arrow indicates the location of the mutation in the gene

The novel mutation site of the *PAH* gene c.60 + 4A > G/p.? is a splicing variant located in intron 1 and was inherited from the father; another mutation site c.158G > A/p.A53H was derived from the mother (Fig. 2).The affected child was diagnosed with mHPA whereas both parents had normal phenylalanine levels. Furthermore, a detailed family history assessment revealed no cases of HPA or other inborn errors of metabolism among the patient’s first-degree and second-degree relatives.

To rule out the possibility of polymorphism, we searched the Single Nucleotide Polymorphism Database, the International HapMap Project Database, and the 1000 Genomes Project Database, and confirmed that this variant is not a polymorphism. Additionally, by reviewing the relevant literature and cross-referencing with the *PAH* gene-specific database (http://www.biopku.org), the Human Gene Mutation Database (HGMD; http://www.hgmd.cf.ac.uk), and the ClinVar database (http://www.ncbi.nlm.nih.gov/clinvar), no previous reports of this c.60 + 4A > G mutation were identified.

**Table 2:** Mutation spectrum and allele distribution of *PAH* genes (*n* = 160)

Index	Nucleotide alteration	Amino acid change	Variant type	Region	Allele frequency	Alleles in HPA		
						mHPA	mPKU	cPKU
1	c.728G>A	p.R243Q	missense	exon7	26.88%	12	17	14
2	c.331C>T	p.R111*	nonsense	exon3	9.38%	3	7	5
3	c.320A>G	p.H107R	missense	exon3	8.13%	12	1	
4	c.158G>A	p.A53H	missense	exon2	7.50%	11	1	
5	c.441 + 2T>A	splicing	splicing	intron4	5.00%	1	2	5
6	c.498C>G	p.Y166*	nonsense	exon5	3.13%	2	1	2
7	c.1068C>A	p.T356*	nonsense	exon11	2.50%		3	1
8	c.1238G>C	p.A413P	missense	exon12	2.50%	1		3
9	c.611A>G	p.T204C	missense	exon6	2.50%			4
10	c.661C>T	p.A221*	nonsense	exon7	1.25%		2	
11	c.1144T>A	p.P392I	missense	exon11	1.88%	2	1	
12	c.782G>A	p.R261Q	missense	exon7	1.88%		3	
13	c.1197A>T	p.V399V	synonymous	exon11	1.25%	1		1
14	c.442-1G>A	splicing	splicing	exon6	1.25%		1	1
15	c.516G>T	p.Q172H	missense	exon6	1.25%	2		
16	c.558_560delGAT	p.T187Gfs	frameshift	exon7	1.25%	1	1	
17	c.694C > T	p.Gln232*	nonsense	exon6	1.25%			2
18	c.721C>T	p.R241C	missense	exon7	1.25%	1	1	
19	c.842 + 1G>A	splicing	splicing	intron8	1.25%	2		
20	c.1068C>G	p.T356*	nonsense	exon11	0.63%		1	
21	c.1066-1G>T	splicing	splicing	exon11	0.63%		1	
22	c.1162G > A	p.V388M	missense	exon11	0.63%		1	
23	c.1199G>C	p.A400T	missense	exon11	0.63%	1		
24	c.1223G>A	p.A408G	missense	exon12	0.63%		1	
25	c.1249T>C	p.T417H	missense	exon11	0.63%		1	
26	c.1252A>C	p.T418P	missense	exon7	0.63%	1		
27	c.1301C>A	p.A434A	missense	exon12	0.63%	1		
28	c.169–2A>G	splicing	splicing	intron2	0.63%			1
29	c.205C>A	p.P69T	missense	exon3	0.63%		1	
30	c.208_210del	p.S70del	frameshift	exon3	0.63%		1	
31	c.250G>T	p.A84T	missense	exon3	0.63%		1	
32	c.284_286del	p.95_96del	frameshift	exon3	0.63%		1	
33	c.2T>A	p.M1?	missense	exon1	0.63%	1		
34	c.353–2A>T	splicing	splicing	exon4	0.63%		1	
35	c.443-1G > A	splicing	splicing	exon5	0.63%		1	
36	c.482T>C	p.F161S	missense	exon5	0.63%			1
37	c.505>T	p.R169C	missense	exon5	0.63%	1		
38	c.59_60delinsCC	p.G20P	missense	exon3	0.63%	1		
39	c.60 + 4A>G	p.?	splicing	intron1	0.63%	1		
40	c.617A>G	p.Y206C	missense	exon6	0.63%			1
41	c.617T>C	p.I224T	missense	exon6	0.63%			1
42	c.706 + 1G>T	splicing	splicing	intron6	0.63%	1		
43	c.722delG	p.A241fs	frameshift	exon7	0.63%	1		
44	c.722G>A	p.A241H	missense	exon7	0.63%		1	
45	c.833C>A	p.T278A	missense	exon7	0.63%		1	
46	c.843 − 10_843-13delTTCT	splicing	splicing	intron2	0.63%			1
47	c.845A>G	p.A282G	missense	exon8	0.63%	1		
48	c.907delT	p.S303fs	frameshift	exon8	0.63%			1
49	chr12q23.2del	/	/	exon6	0.63%			1

### Comparison of mutation site frequencies of *PAH* gene

The high-frequency mutation sites of the *PAH* gene were c.728G >A/p.R243Q (26.88%, 43/160), c.331 C >T/p.R111* (9.38%, 15/160), c.320 A >G/p.H107R (8.13%, 13/160), c.158G >A/p.A53H (7.50%, 12/160), and c.441 + 2T >A/splicing (5.00%, 8/160) (Table 2). The mutation site c.728G >A/p.R243Q is consistent with research results from most regions of China, excluding the north [17–20]. The frequency of c.331 C >T/p.R111* is higher than in other regions [17, 18], while c.320 A >G/p.H107R and c.441 + 2T >A/splicing are high-frequency mutations only identified in this study (Table 3). The top five high-frequency mutation sites of the *PAH* gene in children of different ethnic groups are shown in Table 4. In ethnic minority children, the c.441 + 2T >A mutation was a high-frequency mutation site, and its mutation frequency was significantly higher than that in Han Chinese children (*P* = 0.004). No mutation at the c.1068 C >A site was detected in ethnic minority children.

**Table 3:** Comparison of *PAH* gene mutation types and frequencies across different regions in China

Regions in China	1		2		3		4		5	
	type	frequency	type	frequency	type	frequency	type	frequency	type	frequency
Current research	p.R243Q	26.9%	p.R111*	9.4%	p.H107R	8.1%	p.A53H	7.5%	c.441 + 2T>A	5.0%
Mainland China [18]	p.R243Q	20.2%	p.EX6-96A > G	7.8%	p.V399V	6.3%	p.R241C	6.0%	p.R111*	5.2%
Northwest china [21]	p.A243G	14.0%	p.EX6-96A > G	5.6%	p.T356*	5.0%	p.A413P	4.7%	c.442 -1G > A	4.3%
Central China [20]	p.R243Q	16.1%	p.Y204C	10.1%	p.R241C	7.4%	p.V399V	6.0%	p.R53H	5.4%
Eastern China [17]	p.R243Q	26.0%	p.R241C	14.8%	p.Y204C	9.9%	p.R111*	6.3%	c.442 -1G > A	6.3%
Northern China [14]	p.A243G	17.7%	p.EX6-96G>A	8.3%	p.V399 V	6.4%	p.A53H	4.7%	p.T356*	4.7%
Southern China [19]	p.R243Q	23.2%	p.EX6-96G>A	10.3%	p.IVS7 + 2T>A	8.9%	c.442 -1G > A	6.3%	p.Y356X	4.5%

**Table 4:** High-frequency mutation sites and mutation frequencies of the *PAH* gene in Han Chinese and ethnic minorities

Index	Han Chinese(*n* = 111)				ethnic minorities(*n* = 49)		
	Nucleotide alteration	Amino acid change	Allele frequency		Nucleotide alteration	Amino acid change	Allele frequency
1	c.728G>A	p.R243Q	27.93%		c.728G>A	p.R243Q	24.49%
2	c.331C>T	p.R111X	9.91%		c.320A>G	p.H107R	12.24%
3	c.158G>A	p.A53H	8.11%		c.441 + 2T>A	splicing	12.24%
4	c.320A>G	p.H107R	6.31%		c.331C>T	p.R111X	8.16%
5	c.1068C>A	p.T356*	3.60%		c.158G>A	p.A53H	6.12%

### Relationship between genotype and phenotype in PAHD patients

In the high-frequency mutation sites of the *PAH* gene, specifically c.728G > A/p.R243Q, mHPA accounts for 32.43%. Similarly, in the mutation site c.331C > T/p.R111*, mHPA accounts for 23.07%. However, in mutations c.320A > G/p.H107R and c.158G > A/p.A53H, mHPA accounts for 92.31% and 90.91%, respectively. Compared to children carrying c.728G > A/p.R243Q and c.331C > T/p.R111* mutations, those carrying c.320A > G/p.H107R and c.158G > A/p.A53H (*P* < 0.001) mutations exhibited a higher proportion of mHPA. Among other mutations with allele frequencies exceeding 2%, 75% of children carrying c.1068C > A/p.T356* were diagnosed with mPKU, while 25% were diagnosed with cPKU. All children carrying c.611A > G/p.T204C were diagnosed with cPKU (Table 2).

There were 33 *PAH* genes with null mutations and 63 with missense mutations. Among children with PAHD, 9 cases had the null/null genotype, 15 cases had the null/missense genotype, and 33 cases had the missense/missense genotype. Among patients with cPKU, 52.6% carried at least one null allele, compared with 34.5% in patients with mPKU (Table 5). There was a significant difference in the distribution of the three genotypes between cPKU and mPKU patients (*χ*^*2*^ = 6.83, *P* = 0.033). The frequency of the null/null genotype in cPKU patients was significantly higher than that of the null/missense genotype (*P* = 0.035) and missense/missense genotype (*P* = 0.022) in mPKU patient. There was no significant difference in the distribution of the null/missense genotype and the missense/missense genotype between patients with cPKU and mPKU (*P* = 0.544).

**Table 5:** Relationship between genotype and phenotype in children with PAHD

Phenotype	Mutation types		
	null/null	null/missense	missense/missense
cPKU	7	3	9
mPKU	2	8	19
mHPA	0	4	5

## Discussion

With the increasing screening rate for neonatal inherited metabolic diseases and the widespread use of tandem mass spectrometry detection, children with HPA can now be accurately diagnosed before severe neurocognitive impairment occurs. Timely intervention with a low phenylalanine diet can improve their quality of life and effectively alleviate the mental and economic burden on their families and society. Over the past decade, the government-funded screening program for inherited metabolic diseases in newborns has gradually increased the screening rate in Yunnan Province, maintaining it at over 98%. Yunnan Province, situated in the southwest of China and bordering the South Asian Peninsula, boasts an ethnic composition that markedly differs from other regions in China.In our study, the incidence rate of PAHD was 0.98/10,000, which is higher than the average incidence rate in China (0.68/10,000) [8]. Previous reports suggest that the incidence rate in northern China is higher than that in the south [8], but in this study, the incidence rate of PAHD is close to that in some northern parts of China [18] and higher than that in some southern parts (Xiamen, China, 0.36/10,000) [22], This may be related to the ethnic characteristics that distinguish Yunnan from other regions in China [23]. Moreover, the incidence rate of PAHD in our study was significantly lower than that in European and certain Middle Eastern populations, but higher than that in other countries in the Americas and Asia [12], indicating significant geographical and ethnic differences in the incidence of PAHD.

In the present study, the incidence rate of BH4D stands at 0.16/100,000, which is lower than the overall prevalence rate of BH4D in China (0.38/100,000). Specifically, it’s notably lower than the prevalence rates observed in eastern China (0.59/100,000) and northern China (0.41/100,000). However, it aligns closely with the prevalence rates in southern China (0.16/100,000) and northwestern China (0.17/100,000) [24]. Compared to other countries, the incidence rate of BH4D in our study is lower than that in Brazil (0.21/100,000) [25] but higher than that in Japan (0.06/100,000) [24]. Apart from the genetic background of the populations in these regions, the disparities in screening and diagnostic methods for HPA in these countries may be the primary factors contributing to the differences in incidence rates.

The human *PAH* gene is situated on chromosome 12q22-q24.1, composed of 13 exons and 12 introns, and spans a total length of 90kb [18]. A total of 49 types of *PAH* gene mutations were detected in this study, primarily concentrated in exons 7, 11, 6, and 3, collectively accounting for 63.27% of all mutation sites. In previous studies, *PAH* gene mutations were primarily concentrated in exon 6 [12]. In China, *PAH* gene mutations are mainly found in exon 7 [14, 19], which is consistent with the findings of this study. In our study, the predominant mutation type in the *PAH* gene is missense mutation, aligning with previous research findings, albeit with a higher proportion of splice mutations and nonsense mutations [12, 19].

Globally, the two most prevalent mutations in HPA patients are p.R408W (19.2%) and c.1066-11G >A (6.8%) [12]. In China, p.R243Q is the most common mutation in HPA patients [13], whereas in Spain [26] and the United States [27], the most common mutations are p.Q355_Y356insGLQ and p.R408W, respectively. Consistent with the researches in China, the high-frequency mutation site of the *PAH* gene in this study is c.728G >A/p.R243Q, which is also a prevalent mutation type in regions such as Zhejiang [17] and Shanxi [28] in China. The mutation frequency of c.331 C >T/p.R111* is higher in our study compared to other regions in China [17, 18]. Meanwhile, c.320 A >G/p.H107R and c.441 + 2T >A/splicing are only high-frequency mutations identified in this study, this may be associated with the higher frequency of the c.320 A >G/p.H107R and c.441 + 2T >A/splicing mutation sites in children with PAHD from ethnic minorities in the present study, particularly the frequency of the c.441 + 2T >A/splicing mutation site, which is significantly higher than that in Han Chinese children with PAHD, indicating their regional enrichment in Southwest China.This difference may stem from the unique ethnic demographic structure of Yunnan, such as the unique Y-chromosome haplotypes in indigenous populations [29] and the underlying founder effect [30]. However, high-frequency mutations commonly found in other regions, such as c.611 A >G/p.T204C and c.721 C >T/p.R241C, were only observed in 2.44% and 1.22% of the samples in the present study, respectively [17, 19], indicating that there are differences in the types and frequencies of *PAH* gene mutations across different regions in China. In our study, two children with BH4D were identified as having homozygous mutations in the *QDPR* gene (c.661 C >T/p.R221X) and compound heterozygous mutations in the *PTS* gene (c.286G >A/p.D96N, c.259 C >T/p.P87S), which have also been reported in other studies in China [31, 32].

In the present study, the high-frequency mutation sites of the *PAH* gene, specifically c.728G >A/p.R243Q, c.331 C >T/p.R111*, and c.441 + 2T >A/splicing, were observed in children with cPKU, mPKU, and mHPA. However, a higher proportion of children carrying c.320 A >G/p.H107R and c.158G >A/p.A53H mutations exhibited mHPA, which is consistent with previous researches [10, 18]. This indicates that children with PAH carrying c.320 A >G/p.H107R and c.158G >A/p.A53H mutations exhibit milder clinical symptoms and, for the most part, do not require low phenylalanine diet therapy; Children carrying c.1068 C >A/p.T356* and c.611 A >G/p.T204C mutations require low phenylalanine diet therapy. In previous studies, both genotypes were observed in children with cPKU, mPKU, and mHPA, with mHPA accounting for approximately 14.00% [21]. This may be attributed to the small sample size of this study, and further research with larger sample sizes is warranted to confirm these findings. Our analysis revealed a significant association between *PAH* gene genotype combinations and phenotypic severity in PAHD patients, which provides critical insights into the genetic basis of phenotypic heterogeneity in PAHD. This finding supports the “dosage effect” hypothesis of *PAH* mutations, where the cumulative loss of enzymatic function from biallelic null mutations correlates with more severe hyperphenylalaninemia characteristic of cPKU [33]. Notably, the lack of significant difference between null/missense and missense/missense genotypes suggests that even single null alleles may not sufficiently exacerbate phenotypic severity when paired with missense alleles, possibly due to the compensatory residual activity from missense variants. This observation is reinforced by in vitro functional studies demonstrating that certain missense mutations can retain 3.1% to 21.7% of wild-type PAH activity, which may mitigate the enzymatic loss caused by concomitant null alleles [34]. Researches on global phenotypic distribution indicate that 62.0% of PAHD cases are cPKU [12]. In the Chinese population, the proportion of cPKU stands at 53.0% [18]. However, in the present study, the proportion of cPKU is only 24.8%, which is notably lower than previous studies. This discrepancy may be attributed to the types of gene mutations prevalent in this region.

In four cases of PAHD, only one mutation site was detected. This may be attributed to the fact that some *PAH* gene mutation sites are located in regulatory regions or intron regions, or there may be large deletions that are undetectable using current detection methods [21].

The newly discovered *PAH* gene mutation site c.60 + 4 A >G/p.? is a splicing mutation located in the first intron region. According to the ACMG standards and guidelines for the interpretation of sequence variants [16], it is classified as a “Uncertain significance variants (VUS)” (PM2 + PP3). Multiple bioinformatics tools predict this variant to be potentially pathogenic. Moreover, the splice site prediction tool MaxEntScan showed a substantial decrease in the predicted score from 63 to 40 (a 36.5% reduction). This significant reduction strongly suggests that the mutation is likely to disrupt a critical splice site, leading to abnormal post-transcriptional processing of the *PAH* gene. Consistent with this molecular defect, the patient exhibits elevated phenylalanine levels. Based on this combined genetic and phenotypic evidence, we classify this variant as pathogenic.

Our study has several strengths. We conducted a retrospective analysis of neonatal screening data over the past decade, clarified the data on the *PHA* gene mutation spectrum in the study region, and identified a novel *PAH* gene mutation, which expands the current genetic knowledge. Our study also has some limitations. Since we did not perform follow-up on all screened neonates, those with false-negative screening results could not be included in our database, which may lead to an underestimation of the prevalence rate. Our study lacked an analysis of the relationship between *PAH* mutations and residual PAH activity, which hindered the direct interpretation of the genotype-phenotype relationship. Due to the inability to collect biological samples from more family members of probands with de novo mutations, we were unable to perform systematic segregation analysis for these variants, which weakens the direct evidence supporting the pathogenicity of novel *PAH* gene mutation. Additionally, the small number of BH4D cases limits the generalizability of the study results.

## Conclusions

Neonatal inherited metabolic diseases screening enables early diagnosis and treatment for infants with HPA, thus avoiding neurodevelopmental delays [2]. The incidence rate and gene mutation spectrum of HPA in Yunnan Province, China, differ from those in other regions of China and other countries. This variation is attributed to the geographical and ethnic specificity of the gene mutation spectrum in children with HPA. Our research suggests that different gene mutations may be associated with the disease phenotypes in PAHD, and has identified a new *PAH* mutation gene, thereby enriching the *PAH* gene database. Molecular genetic testing in the present research not only elucidates the pathogenic basis of HPA but also establishes a genetic foundation for optimizing clinical management strategies, including personalized genetic counseling, targeted prenatal diagnostics, and evidence-based therapeutic interventions for affected children.

## Data Availability

The datasets used and/or analysed during the current study are available from the corresponding author on reasonable request.

## Abbreviations

HPA: Hyperphenylalaninemia
PAHD: Phenylalanine hydroxylase deficiency
BH4D: Tetrahydrobiopterin deficiency
mHPA: mild hyperphenylalaninemia
mPKU: mild phenylketonuria
cPKU: classical phenylketonuria
